# Cash Incentives for Institutional Delivery: Linking with Antenatal and Post Natal Care May Ensure ‘Continuum of Care’ in India

**DOI:** 10.4103/0970-0218.45370

**Published:** 2009-01

**Authors:** Chandrakant Lahariya

**Affiliations:** Department of Community Medicine, G.R. Medical College, Gwalior, Madhya Pradesh, India

## Introduction

The obstetrician, paediatrician and public health person alike may be happy by the fact that both maternal mortality ratio (MMR) and infant mortality rate (IMR) are showing signs of improvement in India. The latest census of India estimates on maternal mortality(1) sample registration system (SRS) 2004(2) reports and national family health survey (NFHS)-3(3) findings show that country, although, may not able to achieve the goals set in National Population Policy,(4) is at least making the progress in the right direction. While MMR has come down from 540/lac live births to 301/lac live birth, the IMR has reached at a national level of 57/1000 live births.(3) MMR had almost been static at that level for almost a decade with whole decade on 1990-2000 showing almost no improvement. The latest estimates based upon representative, re-sampled, routine household interview of mortality with Medical evaluation (RHIME) method(1) and gives the most reliable estimates so far, for the period of 1997-2003 and the findings are encouraging. However, unfortunately at this rate, India would not be able to achieve the goal of reducing the MMR by three fourth of 1990 level by 2015 as envisaged in Millennium development Goal (MDG) 5.(5)

Attempts have always been made to understand the mechanism of maternal mortality in India and antenatal care (ANC), skilled birth attendance, and institutional deliveries have been identified as important contributor for reducing MMR. Therefore, the efforts to improve MMR have traditionally focused upon increasing access to health services delivery in India. National Rural Health Mission (NRHM)(6) has reduction in MMR as an important goal and carries many efforts in this direction. One such major strategy under NRHM is to provide cash incentives to the pregnant women, who attend antenatal clinics and opt for institutional deliveries. The scheme is known as ‘Janani Suraksha Yojna’ (JSY).(7) The JSY is the Hindi words which literally mean ‘Pregnant Women Safety Scheme’.

To start with, JSY is a 100% centrally sponsored scheme with provision for cash assistance at delivery and in the post delivery period. The aim of this scheme is reducing maternal and neonatal mortalities by promoting institutional deliveries. This scheme has been modified from earlier National Maternity Benefit Scheme (NMBS) and, is now being run as a part of NRHM. The NMBS was introduced in 2001 to provide nutrition support to pregnant women. Under this scheme below poverty line (BPL) pregnant women are given a onetime payment of Rs. 500/- 8-12 weeks prior to delivery.(8) Following the review of the implementation of this scheme and recommendations from that review, the JSY was planned. The scheme is specifically targeted at scheduled caste/scheduled tribes and poor population. The states have been stratified in low performing states (LPS) and high performing states (HPS) for cash incentives under this scheme with all 8 empowered action group states and Assam, Jammu and Kashmir termed as LPS. JSY has been planned so a women from LPS gets a cash incentive of Rs. 1000-1400 per institutional delivery. Mechanism for disbursement of this money is also elaborately described in the JSY document in practicable manner with good cash incentives for Accredited Social Health Activist (ASHA) too.(9)

There is separate provision of Rs. 250/- for transport in case of emergency with another provision of Rs. 1500/- for caesarian delivery if needed. Furthermore, even after this, if a mother wants to deliver at home, she will be given a cash incentive of Rs. 500/- to meet the on delivery and post delivery expenses.

The scheme has inbuilt mechanism of grievance redress, monitoring and feedback, ensuring that all the aspects of planning, implementation and management are covered under this scheme.(10)

## What is Good in JSY?

JSY is a desired and people centric health program in the country. This is not a first such program in the country and, there have been many similar efforts to improve the maternal survival by providing the cash incentives to the antenatal mothers. However, JSY has evolved from previous experiences and carries the best practices from past. Furthermore, the scheme has inbuilt mechanism of modifications and has also been modified since it was first rolled out in April 2005.(6) There are some changes after the feedback from the ground implementation and the noticeable are in the: removal of age restriction for the benefit in LPS, doing away with the restriction on the order of childbirth, need for BPL or marriage certificate etc. The rationale behind these steps is that a large proportion of deaths occur in the mother aged less than 19 years or the mothers with high birth order. Similarly, getting a BPL certificate is not easy or marriage certificates are not always available. Besides, when the aim of the scheme is to reduce maternal mortality, the aim should be providing care to each mother. The scheme recognizes the differential needs of the states and, is being implemented in stratified manner categorizing the sates on the basis of performance on maternal mortality in LPS and HPS.

The step to give cash incentives for home delivery, although, contrary to increasing the number of institutional deliveries, is still an innovative provision, if a pregnant women for any reason is not able to get institutional care, she should at least get some proper care at home.

## How it can be Improved?

While every issue and rationale of the steps in scheme has elaborately been explained in the ministry document, there are some issues which need consideration and attention:

1. The scheme aims to reduce the maternal mortality in India by increasing the proportion of institutional deliveries. However, it should be remembered that increasing the institutional deliveries is not the only solution for reducing MMR and that's why the target for institutional deliveries is only 80% in National Population Policy document(4) and not 100%. What is more important is identification of high risk pregnancies and giving them priority care, increasing the proportion of deliveries with skilled birth attendants, rapid and timely transport facility for the women in labor to appropriate referral facility, quality of care and accessibility to the health services etc. Therefore, the focus under JSY should be on all aspects of antenatal, postnatal care and the quality of care also rather than on institutional delivery only. Efforts should be made that more number of women receives at least 3 ANC, so to screen them for high risk and subsequently go for institutional delivery.
2. It is a common experience that at present rate of institutional deliveries, hospital beds have 2-3 pregnant mothers on single bed. So, imagine what would happen if the proportion of women who go for institutional delivery increases? Therefore, sufficient attention need to be paid to increase the facilities at hospitals, otherwise poor quality delivery care would be worse than current scenario.
3. Unfortunately, the scheme also considers deliveries at sub centre (SC) as institutional. In the rural health statistics,(11) there are countable numbers of primary health centers with delivery facility excluding the SCs. The inclusion of SC as facility for institutional delivery will inflate the figures and, may give rise to corruption in money distribution under this scheme. The deliveries at SC cannot be managed, in case of complications and these would be as good as home delivery and may even worsen the already eroded faith of general population in health system. However, if we accept the current condition of the SCs good enough for delivery, it may be detrimental for the health system in long run. Besides, ill equipped SC as delivery facility give score to the corruption as deliveries conducted at home may be reported as institutional deliveries at sub centre. Therefore, the efforts should be made to ensure round the clock availability of Auxiliary Nurse Midwife (ANM) at SC, sufficient space and delivery kits at SC for conducting such deliveries. Besides, the efforts should continue to strengthen and equip the Primary Health Centre (PHC) with the facilities for delivery in coming years and sub health centre should work as good referral linking system.
4. JSY has been planned to be too dependent on ASHA. JSY document enlist at least 10 other duties for her in this scheme. The programmers and policy makers have been so fascinated with this worker that she does almost everything, for every scheme under NRHM, on the honorarium basis. In JSY, she does everything for pregnant mother including accompanying her to an institution for delivery. That is asking for too much, when we expect her to work 3-4 hours in a day, and that too, on honorary basis. Her role should be limited to the facilitator and informant, where she may facilitate a pregnant mother and link her to medical officer and institution. The family member of pregnant mother would take care of rest of the things, for the cash incentive. At the same time, this is detrimental to other activities done by ASHA. She would find JSY most lucrative and only high cash driven activity which may lead her paying little attention to other assignments.
5. The cash incentives, which have also been increased since the beginning of the scheme, appears to be sufficient for all the care of mother at the time of delivery and post natal period [Table 1]. However, money disbursement at the time of or after the delivery may serve the purpose partially, as by that time; family would have spent a good amount on number of things. The idea should be to provide some cash to mothers prior to the delivery and the family need not to take any loan from any local money-lender. The author suggests that incentives should be linked to ANC care, delivery, post delivery and mother and infant care events rather than only to the delivery (this would also be a model for continuum of care for both mother and child). In the suggested model, this money should be disbursed as 25% amount for ANC, 50% on delivery and rest 25% linked to mother and infant care (Suggested mechanism in [Table 2].

**Table 1:** Janani Suraksha Yojna in high performing states and low performing states(9,10)

Factor	Low performing states	High performing states
Who is eligible	All pregnant women of any caste, age or income group delivering at health facility	All SC/ST women and, all the women of below poverty line aged 19 and above
Incentives in rural area		
Mothers package	Rs. 1400	Rs. 700
ASHAs package	Rs. 600	Nil
Total	Rs. 2000	Rs. 700
Incentives in urban areas		
Mothers package	Rs. 1000	Rs. 600
ASHAs package	Rs. 200	Nil
Total	Rs. 1200	Rs. 600
Order of delivery	All birth order	Up to two live birth

**Table 2:** Proposed scheme of cash disbursement under Janani Suraksha Yojna in low performing states

Event in pregnancy and afterwards	Mother gets	ASHA gets
Registration within	Rs. 100	Rs. 20
12 weeks		
ANC2 and TT2	Rs. 50	Rs. 10
ANC at 28 weeks	Rs. 25	Rs. 10
ANC at 36 weeks	Rs. 25	Rs. 10
Institutional delivery	Rest of the package	Rest of the package
BCG on birth within	Rs. 50	Rs. 20
one week of delivery		
At 6 weeks of postpartum	Rs. 300	Rs. 50
visit and for DPT1		
DPT3	Rs. 150	Rs. 20
Measles	Rs. 100	Rs. 60
Total	Rs. 800 + rest of the package	Rs. 200 + rest of the package
6. The ASHA getting incentive at the time of delivery only and her performance being adjudged on the basis of institutional deliveries is also not good. This will promote her to pay the whole attention on delivery and not at ANC. Likewise, mother's package; ASHA's package should also be linked to all the stages of mother and infant care. A second issue is that ASHA does not get any honorarium in HPS. She should be given at least some nominal amount in HPS also, whatever it may be, as this is the only significant source of incentive for ASHA in NRHM.
7. There is incidentally, no mention about the facility for ultrasound for these mothers in JSY. A mechanism need to be devised for the institutional arrangement for ultrasound for all those mothers, advised second trimester ultrasound done, to get it done, free of cost. A public private partnership may be a good solution.
8. The strong monitoring and evaluation mechanism under the JSY is required to ensure quality services and to prevent corruption. In the current format, the proportion of deliveries at each sub centre under a primary health centre, the number of JSY beneficiaries who had attended 3 ANC visits, beneficiaries who had received 2 doses of TT would be three good indicators of the health services and may be good factors in preventing forge like reporting home deliveries as institutional deliveries by ANMs/ASHAs to get cash incentives.

## Conclusion

JSY is a good scheme, which is taking proper shape, and reportedly made significant impact on the rate of institutional deliveries, even in poor performing states. The field reports are suggestive of that the high rate of participation by the families in this scheme. The different agencies have reported this to be a good scheme with rare irregularities of corruption and quality of care.(12) These lessons should be utilized for strengthening the scheme implementation. Besides, the schemes with cash incentives should be started with caution and with inbuilt mechanism of phasing out; once the required momentum is achieved. Secondly, the incentive in present scheme can also be utilized for improving ANC coverage and routine immunization, the scheme may be more useful. Thirdly, it is the time that a nationwide evaluation of the impact of this scheme is planned to take necessary corrective measure. The JSY is an example of how well thought government program may make a dent on the health of the people in short period of time. The change in delivery preference behavior in India is a new beginning for the health system in India.

## References

[1] Government of India (2006). Maternal mortality in India: 1997-2003. Registrar General of Census Operation, New Delhi.

[2] Government of India (2006). Sample Registration System bulletin, Registrar General of India, New Delhi.

[3] International Institute of Population Sciences and ORC Macro National Family Health Survey - 3. International Institute of Population Sciences, Mumbai.

[4] Government of India (2000). National Population Policy, Ministry of Health and Family Welfare.

[5] United Nations Millennium development Goals.

[6] Government of India (2006). National Rural Health Mission. Ministry of health and family welfare, Nirman Bhawan, New Delhi.

[7] Government of India (2005). Janani Suraksha Yojana monograph.

[8] Planning Commission (2006). National maternity Benefit Scheme (NMBS). The Planning Commission, New Delhi.

[9] Lahariya C (2008). Janani Suraksha Yojana. Review of Preventive and Social Medicine.

[10] Government of India (2007). Janani Suraksha Yojana: What is new?.

[11] Government of India (2006). Bulletin of Rural Health Statistics in India 2006.

[12] Centre for Social Justice and Health (2007). Review and recommendations on two years of National Rural Health Mission.

